# Keystone Design Perforator Island Flap for the treatment of recurrent pilonidal disease

**DOI:** 10.1093/jscr/rjag569

**Published:** 2026-07-10

**Authors:** Rhys Beaumont, Jason Diab, Assad Zahid

**Affiliations:** Lismore Base Hospital, 60 Uralba Street, Lismore, NSW, 2480, Australia; School of Medicine, Western Sydney University, Building 30, Campbelltown Campus, Narellan Road & Gilchrist Drive, Campbelltown, NSW, 2560, Australia; School of Medicine, Western Sydney University, Building 30, Campbelltown Campus, Narellan Road & Gilchrist Drive, Campbelltown, NSW, 2560, Australia; Campbelltown Hospital, 1 Therry Road, Campbelltown, NSW, 2560, Australia; School of Medicine, University of Notre Dame, 160 Oxford St, Darlinghurst, NSW, 2010, Australia; School of Medicine, University of New South Wales, Wallace Wurth Building (C27), Cnr High St & Botany St, UNSW Sydney, Kensington, NSW, 2033, Australia; School of Medicine, Western Sydney University, Building 30, Campbelltown Campus, Narellan Road & Gilchrist Drive, Campbelltown, NSW, 2560, Australia

**Keywords:** pilonidal sinus, surgical flaps, wound healing, perforator flap

## Abstract

Pilonidal disease is commonly managed with surgical excision; however, recurrence remains frequent, particularly following midline closure. Off-midline flap techniques are increasingly favoured for complex or recurrent disease, as they are associated with reduced wound healing duration and lower recurrence rates. This report describes the case of a 37-year-old obese male with chronic pilonidal disease refractory to multiple prior excisions, who underwent wide local excision and reconstruction with a Keystone Design Perforator Island Flap (KDPIF). Initial recovery was satisfactory, although apical wound dehiscence required subsequent operative washout, ultimately resulting in complete healing. KDPIF reconstruction represents a viable option for recurrent or extensive pilonidal disease and should be considered early in complex cases, with meticulous postoperative care to optimize outcomes.

## Introduction

Pilonidal disease is a suppurative inflammatory condition of the skin and subcutaneous tissue of the sacrococcygeal cleft. It has an estimated incidence of 39.6 per 100 000 [1], occurs two to three times more frequently in men [2] and has a typical age of onset in the second to third decade of life [3]. Predisposing factors include: family history, obesity, prolonged sitting, local trauma, deep natal cleft, increased hair density in the natal cleft [4, 5] as well as stiffer occipital and intergluteal hair [6]. Pilonidal disease presents as recurrent abscesses interspersed with quiescent periods, or as continuously draining sinuses associated with a non-healing wound [7]. Evidence supports an acquired aetiology for pilonidal disease [8], with secondary infection resulting in abscess formation and the development of true sinus tracts [7].

Pilonidal disease is managed surgically, with off-midline techniques demonstrating superior outcomes over both midline closure and healing via secondary intention [9]. Multiple off-midline techniques have been described, including the modified Limberg, modified Karydakis, Bascom cleft lip, V-Y advancement, and Z plasty, yet no single technique has demonstrated clear superiority [10]. This report discusses the use and complications of a Keystone Design Perforator Island Flap (KDPIF), a surgical technique originally developed for the closure of skin defects following skin cancer removal, in a 37-year-old male for the management of chronic and recurrent pilonidal disease.

## Case report

A 37-year-old obese Caucasian male presented to clinic with recurrent gluteal cleft pain and purulent discharge. His medical history included aortic coarctation and chronic pilonidal disease. His relevant surgical history included laparoscopic appendicectomy, multiple excisions of pilonidal sinus tracts and incision and drainage procedures over 5 years. He was referred to a colorectal surgeon for definitive management of his recurrent pilonidal disease.

**Figure 1:**
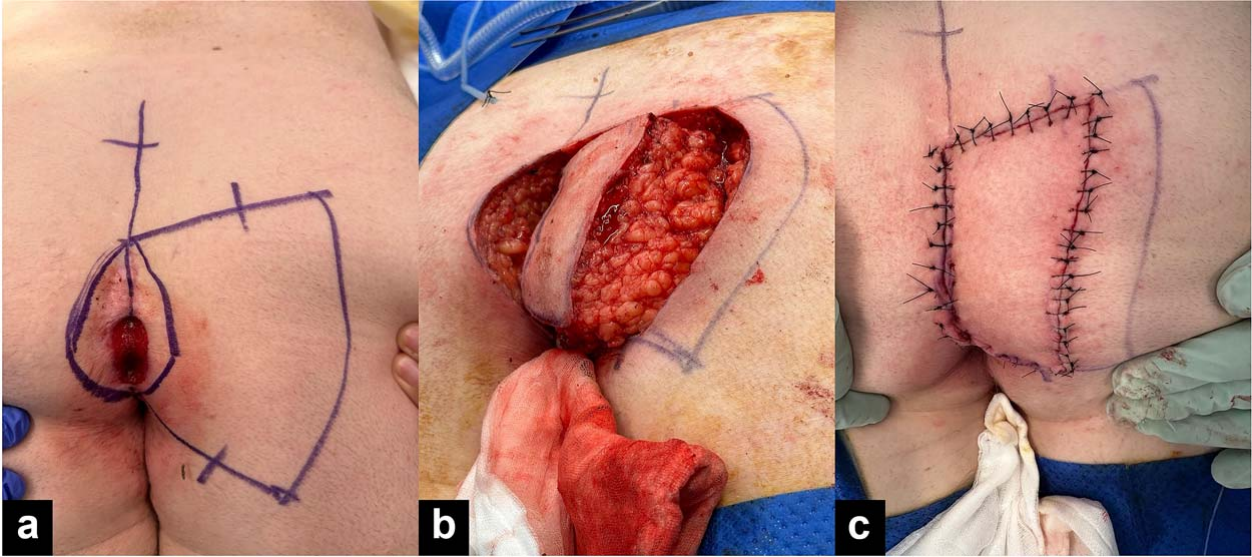
(a) Pre-operative pilonidal sinus and KDPIF set out. (b) Pilonidal sinus excision and mobilization of KDPIF. (c) Immediate postoperative KDPIF.

Given the chronicity, scarred midline tissue and multiple previous failed excisions, he was consented for reconstruction with a KDPIF. The excisional margins, containing the pilonidal sinus and previous scar tissue, were delineated (Fig. 1). A curvilinear trapezoidal shape was designed around the excision of the diseased segment with the right gluteal region selected as the donor site for the flap [11]. The Keystone flap, first described by Behan, resembles a Roman arch based on a random pattern of perforator vessels for the fasciocutaneous flap [12]. The flap was advanced with sound mobility and tension free into the defect, followed by a layered closure with Vicryl and Monocryl skin sutures (Fig. 1). The flap demonstrated immediate perfusion with brisk capillary refill and appropriate tissue colour. A 10Fr blake drain was inserted followed by pressure dressings.

**Figure 2:**
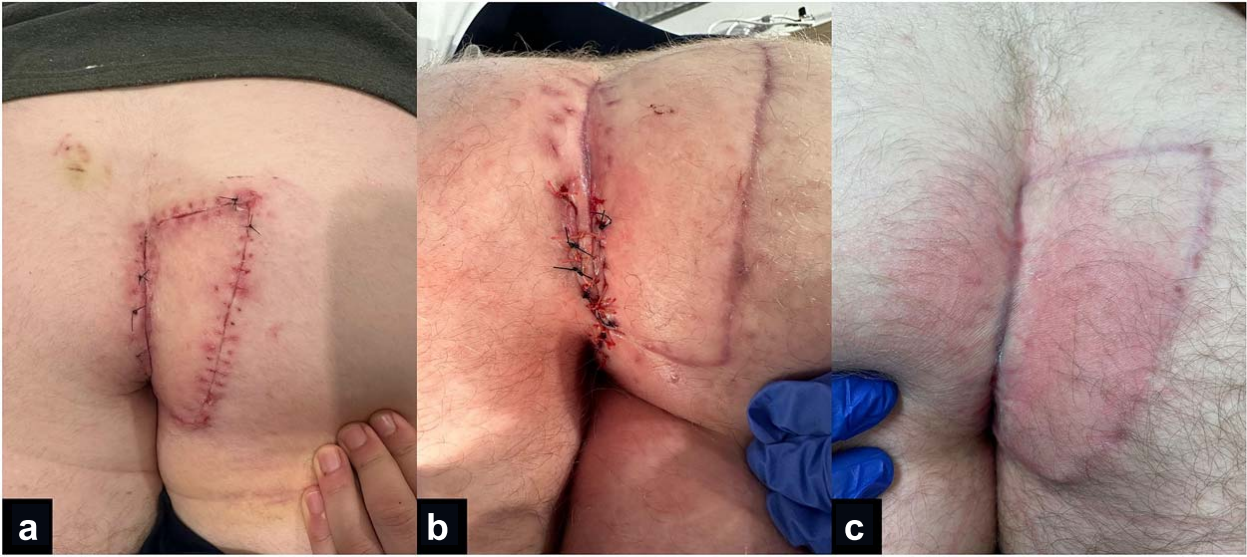
(a) KDPIF 2 weeks post operation. (b) Apical wound dehiscence. (c) Healed KDPIF.

Initial recovery was satisfactory, and the patient was discharged on the third day with postoperative protocol including oral antibiotics, analgesia, aperients, pressure offloading of the buttocks and a ring cushion for support. At 2-week review, a small degree of apical dehiscence was noted inferiorly adjacent to the anus (Fig. 2). The sutures were removed, however premature removal may have contributed to minor wound breakdown. Over the following 2 months, patient compliance with postoperative protocol remained challenging with work commitments, health literacy and psychosocial frustrations impacting everyday living. At 2 months, he presented with a 2 cm superficial cleft dehiscence with purulent discharge requiring operative washout and evacuation of a 7 × 3 × 7 cm cavity requiring a vacuum-assisted closure dressing. Postoperatively the wound improved with outpatient management and ultimately achieved complete healing (Fig. 2). The patient elected to have laser hair removal following surgery.

## Discussion

We present a case of chronic pilonidal disease, treated with a KDPIF, as an alternative management option in carefully selected individuals with sound compliance and understanding. Several patient factors likely contributed to both disease persistence and postoperative complications. The patient’s obesity, sedentary occupation, deep natal cleft, and increased hair density are well-recognized contributors to local maceration and impaired wound healing [4, 5]. Obesity and prolonged sitting are of particular concern as both are associated with increased shear forces across the gluteal cleft, increasing tissue tension and potentially compromising wound integrity. Repeated prior midline incisions further compounded operative complexity by obliterating tissue planes and creating a scarred, poorly perfused natal cleft. Such distorted anatomy must be considered during operative planning impacting upon tissue compliance, perfusion, and tension upon wound closure.

Off-midline surgical techniques demonstrate clear superiority over conventional midline closure, with a systematic review by Cai *et al*. reporting reduced time to wound healing, rate of recurrence, and wound infection rates [9]. Grabowski *et al*. similarly concluded that flap-based reconstruction of any type is preferrable to healing via secondary intention and that midline closure should no longer be considered standard practice [13]. In our case KDPIF was selected specifically due to the need for reliable perfusion within a heavily scarred surgical field [14]. Its perforator vascular supply provides reliable perfusion, while its advancement geometry allowed for lateralization of the scar and partial flattening of the natal cleft, reducing midline tension, and hair accumulation. Compared with rotational or transposition flaps, KDPIF permits adequate coverage of moderate sized defects with relative technical simplicity [15].

Complications following KDPIF reconstruction remain possible despite the favourable outcomes associated with off-midline techniques. Evidence specific to KDPIF use for the treatment of PD is limited; however, reported complications include wound dehiscence, surgical site infection, and rarely flap failure [15]. Risk is increased in individuals with diabetes, obesity, history of smoking, or prior wound dehiscence [10]. In our case, inferior dehiscence was most likely multifactorial, attributable to premature suture removal, local moisture, proximity to the anus, within 1 cm of the sphincter, and postoperative compliance. We advocate for KDPIF use in carefully selected individuals with low risk regarding flaps, longer distance >1 cm from the sphincter, and a clear postoperative protocol. Clinicians with a well-supported wound clinic and experience in flap management and stoma care around the anus is highly useful; we would recommend sutures to remain at least 10–14 days with serial review before removal under trained supervision to minimize risk of dehiscence. We also recommend clear discussion with patients about postoperative care and timeline such as sitting off-side upon the buttocks for at least 2 weeks postsurgery, avoiding resistance training, squatting for long periods, and strenuous activities for at least a month. Laser hair depilation may also be considered to reduce recurrence [16]. This case emphasizes that successful reconstruction depends not only upon flap selection but also consideration of patient social factors, sphincter proximity, and meticulous postoperative wound care.

## Conclusion

In carefully selected individuals with chronic or recurrent pilonidal disease, KDPIF can provide an alternative off-midline closure technique, offering reliable vascular supply for poorly perfused defects. Early consideration of KDPIF in complex or extensive cases, combined with meticulous postoperative wound care and close follow-up, may reduce recurrence and optimize outcomes.
